# Evaluation and management of systemic corticosteroids-induced ocular hypertension in children with non-Hodgkin lymphoma

**DOI:** 10.3389/fped.2022.982224

**Published:** 2022-08-12

**Authors:** Yitian Chang, YuTong Zhang, Zhihua Cui, Xianmei Jin, Yufei Zhao, Lingling Liang, Jian Chang

**Affiliations:** ^1^Department of Pediatric Oncology, The First Hospital of Jilin University, Changchun, China; ^2^Department of Ophthalmology, The Second Hospital of Dalian Medical University, Dalian, China; ^3^Department of Ophthalmology, The First Hospital of Jilin University, Changchun, China

**Keywords:** ocular hypertension, corticosteroid, child, non-Hodgkin lymphoma, intraocular pressure

## Abstract

**Purpose:**

To investigate the effect of systemic corticosteroids (CSs) on ocular hypertension (OHT) and to evaluate the management of OHT in children with non-Hodgkin lymphoma (NHL).

**Methods:**

Medical records of children with NHL treated in our institution between October 2016 and October 2019 were reviewed. The enrolled patients were divided into the mature B-cell lymphoma (MBL) group and lymphoblastic lymphoma (LBL) group based on pathology. Data on routine ophthalmic examinations and management of OHT were recorded.

**Results:**

Of the 54 recruited patients, 38 patients (70.4%) had LBL, and 16 (29.6%) had MBL. Thirty-one patients (57.4%) developed OHT, 24 patients (77.4%) in the LBL group, and 7 (22.6%) in the MBL group. Twelve patients (38.7%) were identified as high responders (10 with LBL and 2 with MBL). Symptomatic patients had a higher mean peak IOP than asymptomatic patients (*p*=0.006). A total of 74.2% of OHT was controlled with antiglaucoma medications (100% in the MBL group *vs*. 66.7% in the LBL group, significant variation, *p* < 0.001). In total, 8 patients (25.8%) underwent tapering of the CSs dose. The duration of OHT was shorter in the MBL group than in the LBL group (*p* = 0.003). No patients were found to have glaucomatous damage or cataracts.

**Conclusions:**

Patients receiving systemic CSs had a higher risk of developing OHT, but the pattern of CSs administration might be a critical factor in the risk and severity of OHT. Tapering of CSs dose should be considered the first line for the management of OHT during high-dose CSs therapy.

## Introduction

Non-Hodgkin lymphoma (NHL) is one of the most common and devastating malignant diseases, especially in young people. With the development of risk-stratification therapy, the 5-year overall survival rates of children with NHL have risen to more than 80–90% over the past 20 years (1). Accordingly, treatment-associated complications have received much more attention for the quality of life of survivors. Corticosteroids (CSs) are almost always used in current chemotherapy protocols, and they play a pivotal role in chemotherapy for childhood NHL (1). In addition to the well-known systemic complications of CSs, CSs-induced ocular hypertension (OHT) has frequently been reported, which can possibly lead to irreversible visual impairment (2). However, to date, most of these studies have been performed on adult patients and topical CSs use (3–6). In contrast, the literature on the effects of systemic CSs use on intraocular pressure (IOP) in the pediatric population has been very limited and controversial (7–10). To our knowledge, no studies have solely examined systemic CSs in children with NHL. Godel et al. reported that systemic CSs use is the least likely route to cause IOP elevation (11). Conversely, we have often clinically observed some children with NHL suffering from OHT during systemic CSs therapy, largely reducing patients' compliance with chemotherapy. Thus, in this retrospective study, we explored the propensity of the systemic administration of high-dose CSs and the role of administration patterns in developing OHT, as well as evaluating the potential risk factors and management of OHT in children with NHL.

## Methods

### Patients

This study was a retrospective and descriptive analytical study. It was approved by the local institutional review board of our hospital, and informed consent was obtained from the patients' parents/guardians. The enrollment criteria were children (younger than 15 years old) diagnosed with NHL and treated in our department between October 2016 and October 2019, whose medical records were reviewed. Based on pathology, the enrolled children were divided into 2 groups: the mature B-cell lymphoma (MBL) group (including diffuse large B-cell lymphoma and Burkett's lymphoma) and the lymphoblastic lymphoma (LBL) group (including precursor T/B-cell lymphoblastic lymphoma). Children with any of the following were excluded: angle anomaly, a history of congenital or developmental glaucoma, a history of glaucoma or high myopia and a history of previous topical/systemic CSs use. Clinical data, including the patient's history of underlying disease and CSs use, ocular examinations, and management of OHT, were recorded.

### Criteria for OHT and routine ophthalmic examinations

OHT was defined as 2 successive IOPs ≥21 mm Hg (or an increase of ≥6 mm Hg from baseline) in either or both eyes. After the commencement of systemic CSs administration, IOP was first measured by Icare rebound tonometry (TAOli, Icare Finland Oy) every 2 to 3 days depending on the severity of OHT. In case of IOP elevation or the reaching of peak IOP, the situation was confirmed once by Goldmann applanation tonometry (900.4.4, Haag-Streit, Koeniz, Switzerland), if possible. All IOP measurements were obtained within the same period of each day between 8 and 10 am. Based on the Armaly and Becker classification, patients with a peak IOP ≥31 mm Hg or a net increase in IOP ≥15 mm Hg in either or both eyes were classified as high responders (12). Additional routine ophthalmic examinations for children with OHT included best-corrected visual acuity, anterior- and posterior-segment evaluation by slit lamp examination, visual field/peripapillary retinal nerve fiber layer thickness and fundus examination. A cup disc ratio >0.4 with asymmetry from the other eye of 0.2 in the cup-to-disc ratio or neuroretinal rim thinning was considered glaucomatous damage (13).

### Administration of systemic high-dose CSs

According to the protocol of NHL-BFM95 (14), patients with MBL received oral dexamethasone (DEX) at a dosage of 10 mg/m^2^/day for 5 days in courses A and B, and 20 mg/m^2^/day for 5 days in course C, for a total of 6 monthly courses.

In the protocol for patients with LBL, CSs were administered as oral prednisolone (PSL) at a dosage of 60 mg/m^2^/day for 28 days with a 2-month break, then a 3-week course of oral DEX at 10 mg/m^2^/day, and finally oral DEX at a dose of 6 mg/m^2^/day for 5 days repeated every 2 months up to a duration of 1.5 years (15).

### Management of OHT

**Figure 1:**
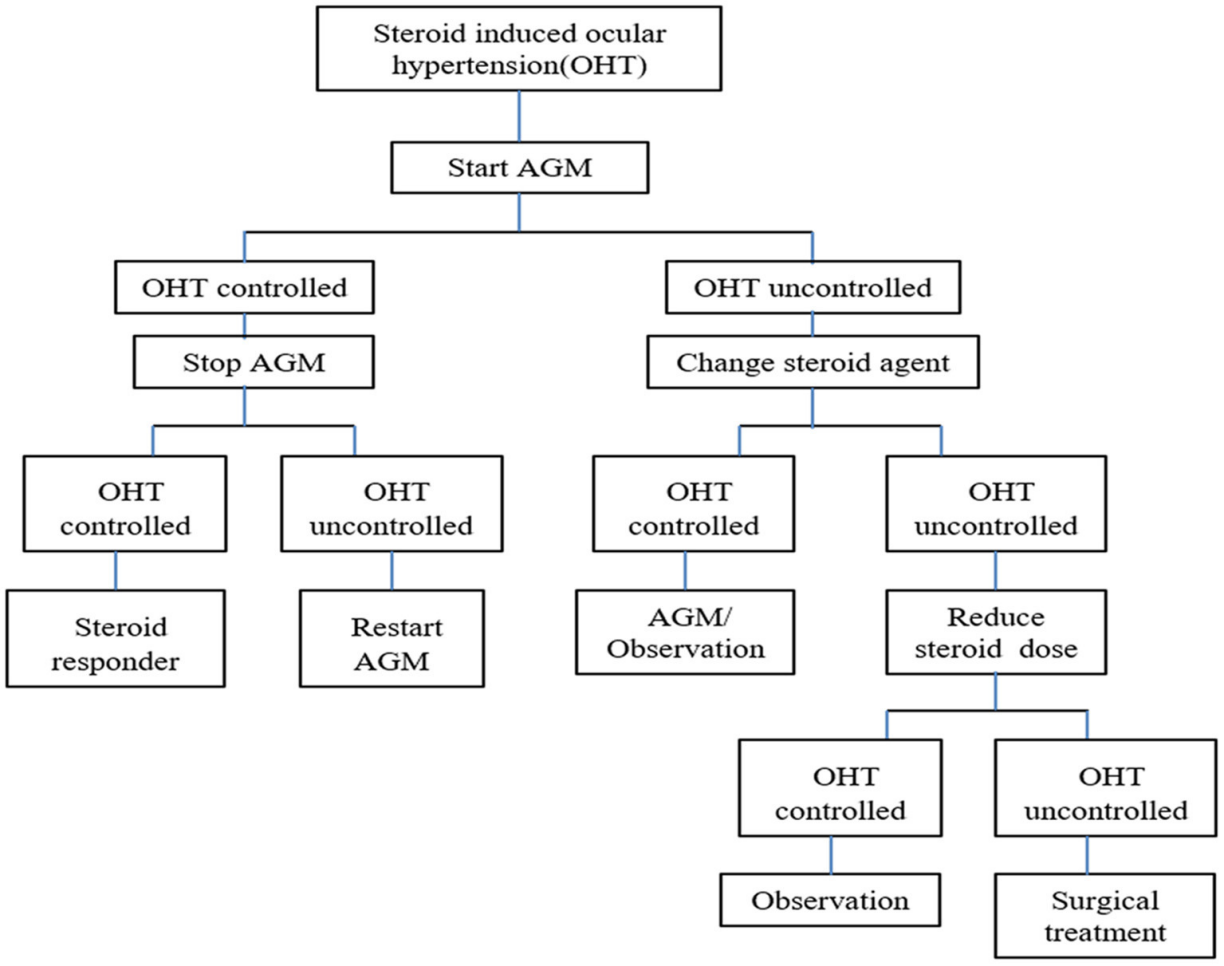
Protocol of management on OHT in flow chart for children with NHL. AGM, antiglaucoma medication; NHL, non-Hodgkin lymphoma.

Patients with an IOP≥21 mm Hg were administered topical antiglaucoma medications (single or combined eye drops), whether with or without any symptoms. IOP <22 mm Hg with or without antiglaucoma medications was considered favorable control. If the elevated IOP could not be controlled ≤ 25 mm Hg by topical combined antiglaucoma medications, the subsequent treatments were switched from DEX to PSL and/or the CSs dose was subsequently tapered (≥two-thirds of the standard dose). The last salvage therapy involved systemic antiglaucoma medication or surgical treatment. The protocol for the management of OHT is depicted in the flow chart (Figure 1).

### Statistical analysis

Statistics were performed using SPSS software, version 12.0.1. The demographic data were assessed by descriptive statistics. Pearson's chi-square test or Fisher's exact test was used to compare possible associations between categorical variables. Linear regression analysis was performed to identify the predictive factors. Parameters among subgroups of patients were compared using analysis of variance. A *p* < 0.05 was defined as statistically significant.

## Results

From October 2016 to October 2019, a total of 58 children treated for NHL (MBL or LBL) in our institute were screened. Four patients were excluded from the analysis due to a lack of routine IOP measurements (2 patients), death at the initial phase of chemotherapy (1 patient) and high myopia (1 patient). As such, fifty-four children (40 boys) were recruited in this study cohort, with a mean age of 8.1 ± 3.75 years old (range, 1.9–15.6 years). Of them, 38 patients (70.4%) had LBL, and the other 16 (29.6%) had MBL. In total, 31 patients (57.4%) developed OHT during or after CSs therapy, 24 patients (77.4%) in the LBL group and 7 patients (22.6%) in the MBL group. In the LBL group, 87.5% of CSs-induced OHT occurred within the first 4 weeks after the commencement of CSs therapy, and only 3 patients presented intermittent OHT during maintenance therapy. Similarly, 85.7% of OHT occurred within the first 3 courses of chemotherapy in the MBL group. Twelve patients (38.7%) were identified as high responders (10 with LBL and 2 with MBL). Seven patients (22.6%) demonstrated symptoms, which were pain in 2 patients, itching in 2, redness of eyes in 3, and watering in 3; notably, only 2 older patients with pain communicated about their complaints. Symptomatic patients had a higher mean peak IOP than asymptomatic patients (36.80 ± 15.56 vs. 30.20 ± 7.20 mm Hg, respectively), and the difference was statistically significant (*p* = 0.006). IOP measurements in the right and left eyes were significantly correlated (r = 0.79, *p* < 0.001), and all statistical analyses were dependent on the right eye data in this study.

Along with lymphoma-related variables, including the serum levels of lactic dehydrogenase (LDH) and ferritin, bone marrow involvement, peripheral white blood cell count and mediastinal mass, all of the potential risk factors for developing OHT were analyzed by binary logistic regression analysis. None of the variables was associated with the risk of OHT, including unfavorable outcomes (relapse or death) (details shown in Table 1).

**Table 1:** Analysis of the risk factors on the development of OHT.

**Factors**		**OHT (+)**	**OHT (-)**	***P***
		***N* (%)**	***N* (%)**	
Age	<10 years	19 (55.9)	15 (44.1)	0.768
	≥10 years	12 (60)	8 (40.0)	
Gender	Male	25 (62.5)	15 (37.5)	0.201
	Female	6 (42.9)	8 (57.1)	
Pathological subgroup	MBL	7 (43.8)	9 (56.2)	0.188
	LBL	24 (63.2)	14 (36.8)	
White blood cell	≥20.0 × 10^9^/L	6 (54.5)	5 (45.5)	1.000
	<20.0 × 10^9^/L	25 (58.1)	18 (41.9)	
Lactate dehydrogenase	≥400 IU/L	14 (48.3)	15 (51.7)	0.144
level (LDH)	<400 IU/L	17 (68.0)	8(32.0)	
Serum ferritin level	≥150 IU/L	14 (66.7)	7 (33.3)	0.394
	<150 IU/L	17 (54.8)	14 (45.2)	
Bone marrow involvement	Yes	17 (54.8)	14 (45.2)	0.658
	No	14 (60.9)	9 (39.1)	
Mediastinal mass	Yes	14 (66.7)	7 (33.3)	0.272
	No	17 (51.5)	16 (48.5)	
Outcome	Survival	26 (53.1)	23 (46.9)	0.064
(Follow-up≥6 months)	Death/relapse	5 (100)	0 (0)	

OHT, ocular hypertension; MBL, mature B-cell lymphoma; LBL, lymphoblastic lymphoma; PSL, prednisolone; DEX, dexamethasone; N, number.

Among the patients with CSs-induced OHT, all OHT-related variables were compared between the MBL and LBL groups. Although patients in the LBL group had a higher prevalence of OHT and included more high responders than those in the MBL group, these differences were not statistically significant. Additionally, no associations were found among peak IOP, time to onset of OHT and cumulative dose of CS at onset of OHT (details shown in Table 2).

**Table 2:** Comparsions of OHT-associated variables on patients with OHT between the MBL and LBL group.

**Factors**	**MBL**	**LBL**	***p* value**
Occurrence of OHT (%)	63.20%	43.80%	0.188
Age (years)	9.14 ± 3.29	8.70 ± 3.49	0.769
Mean time to onset of OHT (days)	35 (5, 66)	14 (8.5, 24.75)	0.925
Mean Peak IOP (mmHg)	28.23 ± 2.65	32.70 ± 10.90	0.077
Mean increase in IOP (mmHg)	12.86 ± 2.61	16.46 ± 10.37	0.134
Duration of OHT after steroid cessation (days)	4.29 ± 1.70	11.00 ± 9.30	0.003
Cumulative dose of PSL equivalent at onset (mg/m^2^)	677 (338, 1,085)	840 (510, 1,200)	0.251
High responder (n/%)	2 (12.5%)	10 (26.3%)	0.162
Control rate of OHT with eye drops	100%	66.70%	<0.001
Average number of anti-glaucoma medications	1.43 ± 0.53	2.17 ± 1.04	0.086

OHT, ocular hypertension; IOP, intraocular pressure; MBL, mature B-cell lymphoma; LBL, precursor T/B-cell lymphoblastic lymphoma; PSL, prednisolone.

In this study cohort, all patients with OHT were administered antiglaucoma medications, and 74.2% of OHT was controlled with single or combined antiglaucoma medications or along with intermittent mannitol use. All (100%) OHT in the MBL group could be controlled with antiglaucoma medications, compared to 66.7% in the LBL group (a significant variation, *p* < 0.001). A 6-year-old boy was found to have a maximal peak IOP of 62 mm Hg, complaining of eye pain. In the LBL group, 8 patients (21%) undertook tapering of their CSs dose, of them, 2 patients who first switched CSs from DEX to PSL finally tapered their CSs dose. Among 8 patients with tapering of CSs doses, 2 patients died (one died from methotrexate-related central nervous system damage and the other from severe pulmonary infection), and there were no relapses. No patients required systemic medication or surgical treatment for OHT.

IOP was normalized in all patients with OHT after cessation of CSs use, and the mean duration of OHT was shorter in the MBL group than in the LBL group, and the difference was statistically significant (*p* = 0.003). Fortunately, in this study, no patients with OHT were identified with glaucomatous damage or cataract by routine ophthalmic examinations during chemotherapy and follow-up (a mean duration of 31.5 ± 17.9 months).

## Discussion

High-dose CSs is an integral part of chemotherapy for childhood NHL. Whether CSs administration is strictly implemented will critically affect the final prognosis (16). A sustained increase in IOP can cause damage to the optic nerve, known as steroid-induced glaucoma (5). Glaucoma is associated with many risk factors, among which elevated IOP is the only known modifiable risk factor. To our knowledge, the other chemotherapeutic agents, in addition to CSs, involved in various protocols for childhood NHL have no known effects on IOP. There is a pressing need to clarify the potential ocular adverse effects from systemic CSs therapy.

In the current study, we found that 57.4% of patients with NHL developed OHT, especially the LBL group, which demonstrated a higher occurrence of OHT (63.2%) and a higher rate of high responders (26.3%) than the MBL group (43.8% and 12.5%, respectively). Although the differences were not statistically significant, they might also be suggestive of the potential impact of patterns of CSs administration in both protocols on OHT. To date, the literature on systemic CSs-induced OHT has remained limited, with varied incidences and inconsistent conclusions (9, 10, 17). de Queiroz Mendonca et al. reported 16.7% steroid responders and 3% high responders among 12 children with hematologic malignant diseases (10). Sugiyama et al. reported 87% steroid responders and 53% high responders among 15 Japanese children (9). The variations were mainly attributed to the heterogeneity of the study designs, definitions of OHT, study populations, and doses and durations of CSs. Regarding the dose and duration of therapy, both largely represent the pattern of administration and are interdependent and inseparable in evaluating the impact of CS on OHT. The pattern of CSs therapy in the LBL group was prolonged continuous administration, which was related to a longer period of therapy and a high cumulative dose of CSs; in contrast, it was intermittent pulse administration in the MBL group that was related to a shorter period of therapy and a lower cumulative dose of CSs. In this study, we found that patients with OHT in the MBL group had a higher control rate of antiglaucoma medication uses and a shorter duration of OHT after CSs cessation than those in the LBL group (*p* < 0.001 and *p* = 0.003, respectively). Thus, the pattern of intermittent pulse administration of systemic CSs might be associated with a more controllable and transient OHT, partly consistent with previous reports (18, 19). However, Godel et al. revealed that CSs-induced OHT did not correlate with the dosage or duration of treatment in their study if IOP rose (20). Huscher et al. first examined patterns of CSs induced side effects and found that glaucoma fit a “threshold pattern” with a threshold dose of 7.5 mg per day in patients on CSs for more than 6 months (21). In our study cohort, this “threshold pattern” might be verified by comparisons of OHT-associated variables, by which no associations were found among increase in IOP, time to onset of OHT, and cumulative dose of CSs at onset of OHT. To date, there have still been no randomized controlled trials to adequately quantify the role of the dose and duration of CSs treatment in developing OHT.

To explore the potential risk factors for OHT, we further analyzed lymphoma-associated variables in this study, including the serum levels of LDH and ferritin, bone marrow involvement, peripheral white blood cell count, etc. None of them was found to be associated with the occurrence of OHT. This result suggested that the factors affecting the severity or prognosis of lymphoma were not involved in the development of OHT. To our knowledge, the present study was the first to evaluate lymphoma-associated variables regarding the risk of OHT (shown in Table 1).

In patients treated with CSs, IOP elevation usually develops within the first few weeks of CSs administration and rarely occurs after a longer period of CSs therapy (2, 22). Similarly, in this study, 87.5% of CSs-induced OHT occurred within 4 weeks after the commencement of CSs use in the LBL group, and 85.7% occurred within 3 courses of chemotherapy in the MBL group. In our study cohort, all patients with OHT were administered antiglaucoma medications, which aimed to prevent possible irreversible visual damage and inevitably affected the actual IOP values. However, the maximal peak IOP still reached 62 mm Hg in a 6-year-old boy with eye pain. Until now, there has been no broad agreement on interventions for OHT. Prada et al. found that young patients with healthy eyes and normal autoregulation of the optic nerve head circulation could tolerate moderate pressure elevations for short periods without nerve damage (23). Therefore, there are various criteria for intervention on OHT in the literature, including no intervention (17), only for symptomatic patients (24), and for patients with IOP ≥25 mm Hg (25). To our knowledge, there have been no reports on the effects of early intervention of OHT on the risk of glaucoma or cataracts. In this study, to explore the long-term ocular side effects of systemic CSs, every patient underwent a routine ophthalmic examination during follow-up (a mean duration of 31.5 ± 17.9 months). However, no patients were found to have glaucomatous damage or cataracts. In contrast, Yan et al. reported 2 patients with glaucoma and 27% posterior subcapsular cataract among 37 Chinese children with systemic autoimmune disease on long-term oral CSs therapy (26). The possible explanations included variations in the detection method and underlying disease, whether or not topical steroids were used, the impact of early intervention, inadequate follow-up and the pattern of CSs therapy. In the latter study, patients received long-term low-dose CSs. Were it used, long-term therapy with low-dose CSs could have resulted in a higher risk of ocular damage than intermittent pulse administration of high-dose CS. However, a conclusion from a meta-analysis by Black et al. showed that there was no statistically significant association between CSs use and the development of glaucoma and cataracts (27). Williamson et al. reported 1 case of glaucoma among 148 CSs users and 1 case among 159 nonusers (28). If IOP rises, discontinuation of CSs therapy is the first line of management. However, it is impossible for patients with NHL. In this study, in addition to the first line of antiglaucoma medications, the subsequent management of OHT included switching from DEX to PSL and tapering of CSs (≥two-thirds of the standard dose). In the LBL group, 2 patents first switched CSs from DEX to PSL along with antiglaucoma medications and finally tapered CSs, suggesting that OHT could not be controlled by switching CSs during high-dose CSs therapy. In total, OHT in 8 patients was controlled with tapering CSs along with antiglaucoma medications without surgery or systemic medications; fortunately, no patients had unfavorable outcomes owing to tapering of their CSs dose. Therefore, during high-dose CS therapy, tapering of the CSs dose should be considered the first line of management of OHT. However, the impact of tapering the CSs dose on prognosis in patients with NHL must be further tested.

The management of childhood NHL is a complex process that is not only related to controlling underlying malignant diseases but also to preventing various treatment-related complications. Clinically, it is easy for a pediatric oncologist to neglect CSs-induced OHT because patients with NHL always have severely poor systemic conditions and have no complaints of ocular symptoms. Most reports showed that CSs-induced OHT was absent of symptoms (3, 9, 10), but there were 7 symptomatic patients (22.6%) in this study, among whom only 2 patients communicated their complaints. Therefore, much more attention should be given to patients' ocular symptoms during CSs treatment, such as redness, watering, photophobia and strabismus, which are easily confused with eyestrain.

There are some limitations in the current study. First, it was a retrospective, observational study, and the baseline IOP was not measured at the commencement of CSs therapy in each patient due to the patients' poor conditions. Consequently, in some patients, OHT and high response were identified only by peak IOP ≥21 mm Hg and 31 mm Hg without increases in IOP ≥6 mm Hg and 15 mm Hg, respectively. This issue inevitably decreased the occurrence of OHT and high responders. Second, the number of study patients was not sufficiently large, and the rare occurrence of ocular adverse effects might not have been captured. Third, the follow-up duration was inadequate. The strength of the present study was that we examined the pattern of CSs therapy and evaluated their effects on the development of OHT through two different protocols for pediatric NHL and simultaneously evaluated our management procedure on OHT in NHL children.

In conclusion, this study provided strong evidence that systemic high-dose CSs can cause significant OHT, with more high responders and a pattern of CSs therapy having an impact on the severity and duration of OHT. Tapering of CSs doses should be considered the first line of management for OHT in NHL children. In addition, these findings raise the need for future well-designed observational studies for further exploration of these undetermined questions.

## Data availability statement

The original contributions presented in the study are included in the article/supplementary material, further inquiries can be directed to the corresponding author.

## Ethics statement

The studies involving human participants were reviewed and approved by the First Hospital of Jilin University. Written informed consent to participate in this study was provided by the participants' legal guardian/next of kin.

## Author contributions

YC, YZhan, and JC made substantial contributions to design of the work, drafted the manuscript, and agree to be accountable for all aspects of the work in ensuring that questions related to the accuracy or integrity of any part of the work are appropriately investigated and resolved. ZC and XJ made substantial contributions to design of the work and drafted the manuscript. YZhao and LL made substantial contributions to the design of the work revised the manuscript critically. All authors have read and approved the manuscript.

## Funding

Project supported by the Jilin Provincial Department of Finance, China (Grant No. JLSWSRCZXL2021-083) fund by YZhan.

## Conflict of interest

The authors declare that the research was conducted in the absence of any commercial or financial relationships that could be construed as a potential conflict of interest.

## Publisher's note

All claims expressed in this article are solely those of the authors and do not necessarily represent those of their affiliated organizations, or those of the publisher, the editors and the reviewers. Any product that may be evaluated in this article, or claim that may be made by its manufacturer, is not guaranteed or endorsed by the publisher.

## Abbreviations

CSs: systemic corticosteroids
Cis: confidence interval
DEX: dexamethasone
IOP: intraocular pressure
LBL: lymphoblastic lymphoma
LDH: lactic dehydrogenase
MBL: mature B-cell lymphoma
NHL: non-Hodgkin lymphoma
OHT: ocular hypertension
PSL: prednisolone.
