# Corrigendum: Evaluation and management of systemic corticosteroids-induced ocular hypertension in children with non-Hodgkin lymphoma

**DOI:** 10.3389/fped.2022.1034111

**Published:** 2022-11-02

**Authors:** Yitian Chang, YuTong Zhang, Zhihua Cui, Xianmei Jin, Yufei Zhao, Lingling Liang, Jian Chang

**Affiliations:** ^1^ Department of Ophthalmology, The Second Hospital of Dalian Medical University, Dalian, China; ^2^ Department of Pediatric Oncology, The First Hospital of Jilin University, Changchun, China; ^3^ Department of Ophthalmology, The First Hospital of Jilin University, Changchun, China

**Keywords:** non-Hodgkin lymphoma, ocular hypertension, corticosteroid, child, intraocular pressure

A corrigendum on Evaluation and management of systemic corticosteroids-induced ocular hypertension in children with non-Hodgkin lymphoma By Chang Y, Zhang Y, Cui Z, Jin X, Zhao Y, Liang L and Chang J. (2022) Front. Pediatr. 10: 982224. doi: 10.3389/fped.2022.982224

***Original version of affiliation(s)***

**(the correct version of the affiliation(s):**

**Yitian Chang^1,2†^, YuTong Zhang^1†^, Zhihua Cui^3^, Xianmei Jin^1^,**

Yufei Zhao^1^, Lingling Liang^3^ and Jian Chang^1*^

**^1^Department of Pediatric Oncology, The First Hospital of Jilin University, Changchun, China,**

**^2^Department of Ophthalmology, The Second Hospital of Dalian Medical University, Dalian, China,**

**^3^Department of Ophthalmology, The First Hospital of Jilin University, Changchun, China)**

**Incorrect Affiliation**

In the published article, there was an error in the order of affiliations 1 and 2. The corrected affiliation list appears below.

Yitian Chang^1†^, YuTong Zhang^2†^, Zhihua Cui^3^, Xianmei Jin^2^, Yufei Zhao^2^, Lingling Liang^3^ and Jian Chang^2*^

^1^Department of Ophthalmology, The Second Hospital of Dalian Medical University, Dalian, China,

^2^Department of Pediatric Oncology, The First Hospital of Jilin University, Changchun, China”, it should be “Yitian Chang^1,2†^, YuTong Zhang^1†^, Zhihua Cui^3^, Xianmei Jin^1^, Yufei Zhao^1^, Lingling Liang^3^ and Jian Chang^1*^

^1^Department of Pediatric Oncology, The First Hospital of Jilin University, Changchun, China,

^2^Department of Ophthalmology, The Second Hospital of Dalian Medical University, Dalian, China,”.

The authors apologize for this error and state that this does not change the scientific conclusions of the article in any way. The original article has been updated.

